# Enhancement of prefrontal functional connectivity under the influence of concurrent physical load during mental tasks

**DOI:** 10.3389/fnhum.2024.1500470

**Published:** 2024-12-24

**Authors:** Shan Cheng, Lin Cong, Duoduo Hui, Chaolin Teng, Wenbin Li, Jin Ma

**Affiliations:** ^1^Department of Aerospace Medical Equipment, School of Aerospace Medicine, Air Force Medical University, Xi’an, Shaanxi, China; ^2^Department of Aerospace Hygiene, School of Aerospace Medicine, Air Force Medical University, Xi’an, Shaanxi, China

**Keywords:** mental workload, functional near-infrared spectroscopy, functional connectivity, cognitive task, physical load

## Abstract

**Backgrounds:**

Functional near-infrared spectroscopy (fNIRS) is widely used for the evaluation of mental workload (MWL), but it is not yet clear whether it is affected by physical factors during cognitive tasks. Therefore, the combined effects of physical and cognitive loads on hemodynamic features in the prefrontal cortex were evaluated.

**Methods:**

Thirty-three eligible healthy male subjects were asked to perform three types of cognitive tasks (1-back, 2-back and 3-back). Concurrently, isotonic contraction aerobic exercise of the left upper limb was added. During this compound task, fNIRS signals, workload perception and task performance were recorded. Based on the oxyhemoglobin concentration, Pearson’s correlation coefficient (CORR), coherence value (COH) and the phase-locking value (PLV) were calculated to reflect FC among eight channels.

**Results:**

On the basis of effects of cognitive tasks, the concurrent physical activities would further increase National Aeronautics and Space Administration Task Load Index score (*p* < 0.05) and decrease task performance (*p* < 0.05). The fNIRS-based results showed that cognitive and physical loads had significant interaction effects on CORR (*p* < 0.05), COH (*p* < 0.05), and PLV (*p* = 0.010), while their main effects were not significant. The results of different channel pairs suggested that the functional connectivity between the right dorsolateral prefrontal cortex and the bilateral orbital frontal cortex was significantly enhanced under the combined effects of high physical and high cognitive load.

**Discussion:**

From the perspective of prefrontal functional connectivity, this study supports measurable effects of physical factor on operators’ mental load. The results provide a reference for the real-time (or online) assessment of the MWL level in the natural environment.

## 1 Introduction

With the development of automation technology, human beings have been liberated from manual work to become task supervisors and decision makers, and need to utilize more and more cognitive resources. Mental workload (MWL) thus becomes a risk factor that may induce human errors, especially in piloting airplanes, driving vehicles, air traffic control and other complex operations. The levels of MWL can be adjusted by changing the difficulty or complexity of cognitive tasks (van Weelden et al., 2022). A relatively high or low MWL can cause the operator to ignore critical information (Durantin et al., 2014). When it is too high, the operator may no longer be competent for the current task requirements or may suffer from mental fatigue, damaging work performance and even causing accidents (Gateau et al., 2018). Similarly, when being in a low MWL state, their alertness decreases and their attention is distracted, or they show disinterest, which further leads to adverse consequences. Therefore, it is necessary to measure and evaluate the operator’s MWL in real time in particular for a pilot, driver and air traffic controller. Current methods for assessing MWL can be broadly categorized into subjective and objective approaches. These include subjective scales, behavioral indicators such as task performance and eye-tracking characteristics, skin conductance levels and physiological signals (Baldauf et al., 2009). However, each approach has its advantages and disadvantages, since the use of subjective scales requires frequent interruptions of tasks to complete the assessments, resulting in low temporal resolution and an inability to monitor in real time and behavioral indicators, such as task performance, face similar limitations. In contrast, objective detection methods based on physiological signals, such as EEG, and ECG can be performed concurrently with tasks, offering high temporal resolution and making them key for real-time monitoring during tasks. Functional near infrared spectroscopy (fNIRS) has the advantages of low cost, portability, good anti-interference, high temporal and spatial resolution, and is widely used for the evaluation of mental load. Based on the absorption specificity of oxyhemoglobin (HbO) and deoxyhemoglobin (HbR) for near-infrared spectroscopy at 700–900 nm, fNIRS can reflect neurocognitive activity level by detecting the relative concentration changes in HbO and HbR. Studies have confirmed that an increase in cognitive load leads to a raised HbO concentration and a decrease in HbR concentration in the cognition related prefrontal cortex (PFC) (Molteni et al., 2008; Molteni et al., 2012; Taylor et al., 2020), indicating this brain region is activated (Saikia et al., 2021). Under the influence of MWL caused by consecutive interpreting tasks, the mental demand score of the National Aeronautics and Space Administration Task Load Index (NASA-TLX) scale was shown to be positively correlated with the activation levels in three brain regions (inferior frontal gyrus, middle temporal gyrus, and inferior temporal gyrus), and negatively correlated with job performance (interpretation quality) (Yan et al., 2024). Li et al. confirmed when subjects completed multiple tasks under a high mental load, that the levels of subjective task load (NASA-TLX) and prefrontal activation measured by fNIRS increased significantly, and there was a significant positive correlation between them (Li et al., 2022). In addition, it has been reported that the prefrontal activation level decreased when the mental overload induced by the highest difficulty level of simulated vehicle driving occurred. The finding implies the existence of a quadratic model of MWL (Durantin et al., 2014). McKendrick et al. pointed out that in the case of a low cognitive load and cognitive overload during the same spatial memory task, the workload transition stage induced significant changes in the cubic functions related to HbO in the left dorsolateral prefrontal cortex (DLPFC) (Brodmann’s Area 46) and working memory load (McKendrick and Harwood, 2019). It has been shown that fNIRS is sensitive to changes in MWL, and that increases of MWL enhance the PFC activation. When MWL reaches the overload state, the degree of activation may be attenuated.

Compared with simulated flight conditions, pilots operating under real conditions might make more errors when performing cognitively demanding tasks, with greater PFC activation measured by fNIRS. This suggests that investigations of MWL assessment in the natural environment is necessary (Gateau et al., 2018). Brain functional characteristics are often affected by various non-cognitive factors (e.g., stress, physical load (PL) and radiation) in the context of actual work (Liang et al., 2023). For example, in a real flight simulator task, Causse et al. confirmed that the increasing effect on the HbO concentration was more obvious in brain regions involving the executive control network (e.g., DLPFC and lateral parietal region) when combined with social stressors (Causse et al., 2024). The combined exposure of electromagnetic radiation and low-frequency noise significantly extended the correct reaction time of n-back cognitive tasks, and increased the relative HbO concentration and its mean *β* value in DLPFC (Liang et al., 2023). Furthermore, compared with an electroencephalogram, fNIRS has better robustness to motion artifacts and environmental noise, and is more suitable for application in real environments (Gateau et al., 2018). Under simulated driving conditions, Li et al. showed that the use of fNIRS, as a tool for real-time evaluation and monitoring of urban rail transit drivers’ mental load, was feasible and reliable (Li et al., 2019). Gateau et al. developed an online passive fNIRS-based brain-computer interface to distinguish two levels of working memory load in highly ecological flight background task, with a classification accuracy of 80%, specificity 72%, and sensitivity 89% (Gateau et al., 2015; Gateau et al., 2018). These results verified the feasibility of passive monitoring of MWL based on fNIRS in real and complex situations. Therefore, from the perspective of practical application, fNIRS technology can be adopted to study evaluation of MWL under the interaction effects of complex factors.

PL, as a factor opposite to mental load, also affects the assessment of MWL. In the study of Albuquerque et al., PL caused by treadmill and bicycle riding increased the subjective mental demand (NASA-TLX) of the subjects when completing multiple cognitive tasks (Albuquerque et al., 2020). Our previous studies also showed similar changes of task-load perception on cognitive tasks. The combined effect of cognitive load and PL also further altered the ECG characteristics, showing increased sympathetic excitability and decreased parasympathetic tone (Cheng et al., 2024). On the basis of our previous studies, we explored the changing characteristics of hemodynamics in PFC under these combined effects. Presently, there appears to be some variation in the results based on fNIRS. One study reported that compared with 15% maximal voluntary contraction of isometric grasping, the mental task performance at the 30% level was significantly reduced, and PFC activity increased significantly (Mandrick et al., 2013). The activation of DLPFC during an implicit cognitive reappraisal task was also enhanced after acute exercise (Zhang et al., 2021). Other studies also indicated that acute aerobic exercise reduced activation of the PFC (Zheng et al., 2022). Thus, in order to explore the interaction between MWL and PL on PFC activation, the present study design combined conditions of different cognitive and physical levels. PFC activation was explored from fNIRS-based function connectivity modes under these combined tasks.

## 2 Materials and methods

### 2.1 Subjects

Thirty-three male subjects were recruited, ranging in age from 20 to 32 years, with an average age of 23.4 ± 3.5 years. All subjects were right-handed and had no fractures, muscle injury or other disease that may have affected limb movements in the last 3 months. According to the self-reports, they did not drink alcohol or take sleeping pills before the experiment. This study was in line with the Declaration of Helsinki and was approved by the Ethics Committee of the Air Force Medical University. Prior to a test, subjects were informed of the experiment content and procedure, and voluntarily signed written informed consent.

### 2.2 Design of cognitive and physical loads

**Figure 1:**
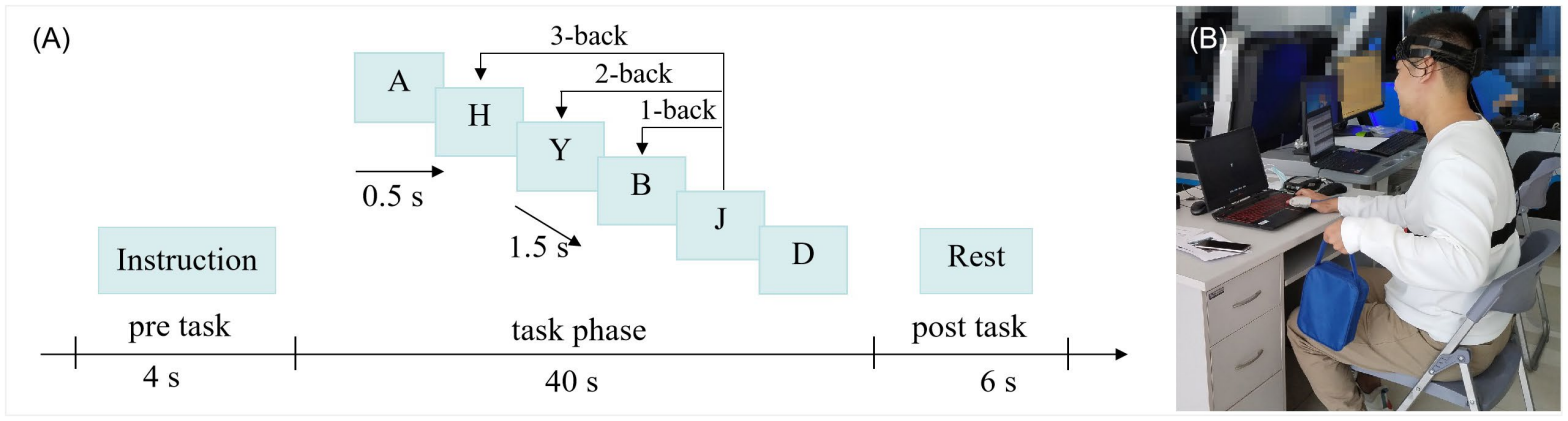
Design of mental load and physical load. In **(A)**, one block takes 50 s, including pre-task indicator (4 s), task phase (40 s), and a resting period (6 s). During the task, the letter appeared for 0.5 s, and the interval was 1.5 s. In **(B)**, isotonic contraction of the left upper limb was adopted and the frequency of contraction was consistent with the frequency of the n-back letters.

N-back working memory task has been confirmed by previous studies to induce MWL (Karthikeyan et al., 2021; Wriessnegger et al., 2021; Cheng et al., 2024). Three MWL states were induced by different difficulties of n-back tasks (1-back, 2-back and 3-back), which have been previously described in detail (Cheng et al., 2024). Subjects were asked to keep their attention on the letter presented in the center of a computer screen and determine whether it was the same as the n previous letter (*n* = 1, 2, 3). If so, they quickly pressed the corresponding key on a keyboard. Each block consists of 20 letters (5 target letters), and each n-back condition was comprised of 6 blocks, which took about 5 min to complete (Figure 1A).

During n-back cognitive tasks, subjects performed physical tasks by lifting heavy objects with the left upper limb in isotonic contraction mode (Figure 1B), and the contraction frequency was consistent with the frequency of n-back letters (2 s/time). Three levels of PL were designed in this experiment, which were none physical load (NP, 0 kg), medium physical load (MP, 3 kg) and high physical load (HP, 5 kg). The details are given in our previously published work (Cheng et al., 2024).

### 2.3 MWL evaluation

#### 2.3.1 Behavioral measurements

Behavioral indicators include subjective feelings and task performance. Subjects’ feelings during experimental periods were assessed by the NASA-TLX, which has been widely used in previous research (Albuquerque et al., 2020). The NASA-TLX scale contains 6 dimensions with 21-points at each namely: mental demand; physical demand; time demand; self-performance; effort level; and frustration level. Task performance refers to the ability to identify target letters during n-back tasks, including: (1) mean reaction time (MRT); standard deviation of reaction time (SDRT); maximum reaction time (maxRT); and (2) the rate of correct number (CNR) and rate of wrong number (WNR) (Cheng et al., 2024).

#### 2.3.2 Hemodynamic features in the PFC

**Figure 2:**
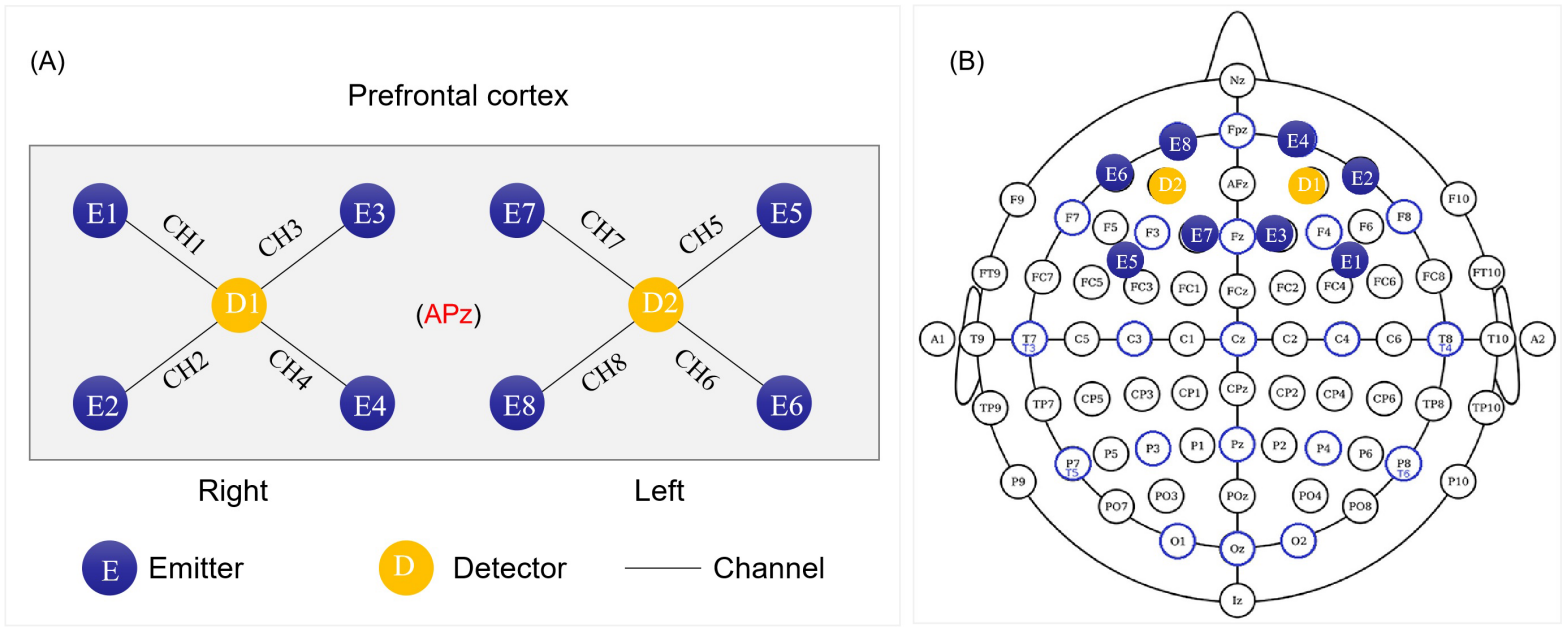
Schematic diagram of functional near-infrared spectroscopy probe layout **(A)** and anatomical position **(B)**. CH1, right dorsolateral prefrontal cortex; CH2, right ventrolateral prefrontal cortex; CH3 and CH4, right orbital frontal cortex; CH5, left dorsolateral prefrontal cortex; CH6, left ventrolateral prefrontal cortex; CH7 and CH8, left orbital frontal cortex.

Cerebral hemodynamic changes in the PFC were recorded by a portable fNIRS system (OctaMon, Artinis, Netherlands), at a sampling frequency of 10 Hz. A 2 × 4 headgear layout was designed, including 8 effective test channels composed of 8 light emitters (each contained 2 light sources of 760 nm and 850 nm) and 2 light receivers (Figure 2A). In the experiment, the 10–20 EEG system was used as the positioning standard to locate the brain functional area, and the APz site was located in the center of the light source detector distribution (Figure 2B). According to Brodmann’s anatomical regional system of the cerebral cortex, Channels 1–4 covered the right PFC (CH1: right DLPFC; CH2: right ventrolateral prefrontal cortex (VLPFC); CH3 and CH4: right orbital frontal cortex (OFC)). Channels 5–8 corresponds to the left prefrontal area (CH5: left DLPFC; CH6: left VLPFC; CH7 and CH8: left OFC).

Matlab 2022b and the homer 2 toolbox were used to preprocess the raw blood oxygen signal. Generally, the fNIRS signal is mainly affected by motion artifacts and physiological interference. The main source of movement artifacts is the physical activity of subjects, such as blinking and head shaking. Physiological interference causing fluctuations of the blood oxygen index mainly comes from physiological activities, such as the heartbeat, breathing, blood pressure fluctuations, blood flow, capillary activity and spontaneous low-frequency vibration. Band pass filtering at 0.01–0.10 Hz was used to remove high frequency noise, baseline drift and physiological interference. Then, the modified Lambert–Beer law was applied to convert the light intensity signal of 2 wavelengths per channel into the relative concentration changes of HbO and HbR. Finally, 8 channels were formed into 28 pairs of connected edges (Table 1), and the indicators related to static functional connectivity (FC) between channels (edges) were calculated. In the study of Chao et al. (2021) three methods were used to calculate FC with detailed calculation processes provided including correlation coefficient (CORR), coherence value (COH) and phase-locking value (PLV) (Chao et al., 2021). In order to make the distribution of correlation coefficient more normal and enhance statistical effectiveness, the CORR value was transformed using the Fisher z method.

**Table 1:** The corresponding relationship between edges and channel pairs.

Edge	Channel-pair	Edge	Channel-pair	Edge	Channel-pair	Edge	Channel-pair
01	CH1-CH2	08	CH2-CH3	15	CH3-CH5	22	CH4-CH8
02	CH1-CH3	09	CH2-CH4	16	CH3-CH6	23	CH5-CH6
03	CH1-CH4	10	CH2-CH5	17	CH3-CH7	24	CH5-CH7
04	CH1-CH5	11	CH2-CH6	18	CH3-CH8	25	CH5-CH8
05	CH1-CH6	12	CH2-CH7	19	CH4-CH5	26	CH6-CH7
06	CH1-CH7	13	CH2-CH8	20	CH4-CH6	27	CH6-CH8
07	CH1-CH8	14	CH3-CH4	21	CH4-CH7	28	CH7-CH8

CH, channel; CH1, right dorsolateral prefrontal cortex; CH2, right ventrolateral prefrontal cortex; CH3 and CH4, right orbital frontal cortex; CH5, left dorsolateral prefrontal cortex; CH6, left ventrolateral prefrontal cortex; CH7 and CH8, left orbital frontal cortex.

In the research of Peng et al. (2021), the Pearson correlation coefficient was used to study the dynamic interaction between regions during cortical activity. These methods for calculating FC are fundamentally similar and the present study was conducted with reference to their methods.

### 2.4 Experimental procedure

**Figure 3:**
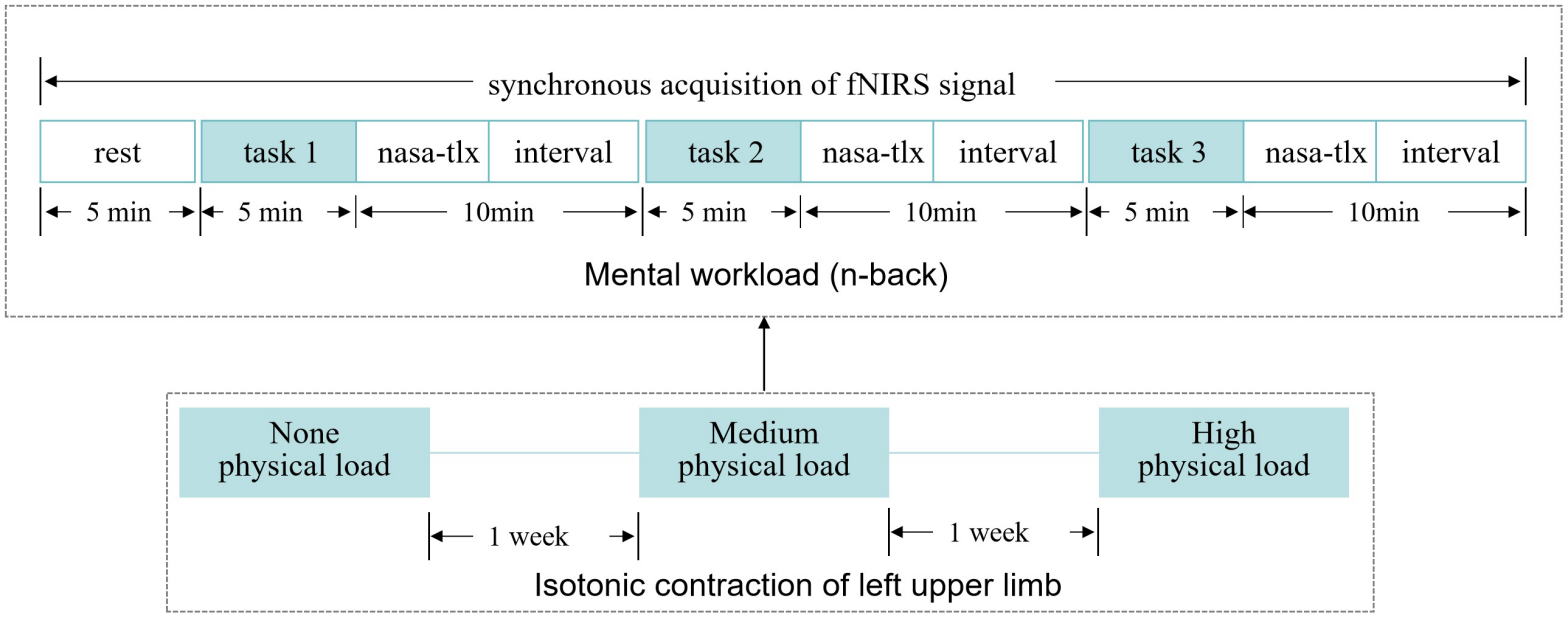
Experimental procedure. Task 1, task 2 and task 3, respectively, refer to each difficulty of an n-back task. In order to minimize practice effects, test sequences of 1-back, 2-back and 3-back were balanced; the interval between them was 10 min. fNIRS, functional near-infrared spectroscopy.

This study was designed with two independent variables: physical workload (with three levels: NP, MP, HP) and MWL (with four levels: resting, 1-back, 2-back, 3-back). A repeated measures design was employed, requiring participants to complete n-back cognitive tasks under each of the three physical workload conditions. The interval between different physical workload conditions was 1 week. Before each experiment, participants’ foreheads were cleaned with alcohol wipes, and an fNIRS measurement headband was worn. Participants then practiced a single 2-back task. Once familiar with the experimental procedure and tasks, they proceeded to the formal testing phase. Initially, 5 min of PFC activity were recorded during a resting state, followed by participants completing cognitive tasks according to a predetermined n-back test sequence with three levels of difficulty (to balance the order of difficulty and minimize practice effects). Immediately after each task, participants evaluated their subjective workload using the NASA-TLX scale. A 10-min interval was set between cognitive tasks, and fNIRS signals were collected throughout the entire experimental process (Figure 3).

### 2.5 Statistical analysis

In this study, cognitive load and PL were, respectively, set as the intra-group factor and inter-group factor, with their main and interaction effects studied. Behavioral data (subjective scale and task performance) was analyzed using SPSS v23.0 software. If the data conformed to a normal distribution and homogeneous variance, analysis of variance (ANOVA) for repeated measurement and the least significance difference (LSD) of *post hoc* tests were used to analyze the overall difference and pairwise comparison between three PL levels. If not, the data were analyzed by Friedman’s 2-way ANOVA by ranks and multiple comparisons in a nonparametric test. And the FC indices based on fNIRS were analyzed in unit of edges by the Matlab 2022b software and ANOVA for repeated measurement. The *p*-values of all edges are corrected with the false discovery rate to control the multiple comparison problem. If the main effect and (or) interaction effect are significant, the post-hoc test were further performed, and its *p*-value would be corrected by using the Bonferroni method. *p* < 0.05 was deemed to be the level of a statistically significant difference. The sample size calculated with G*Power (Version 3.1.9.7) was 30, and the key parameters was effect size 0.25, *α* error probability 0.05 and power (1-*β* error probability) 0.8. In this study, 33 subjects were selected, who met the eligibility criteria.

## 3 Results

### 3.1 Behavioral changes

**Figure 4:**
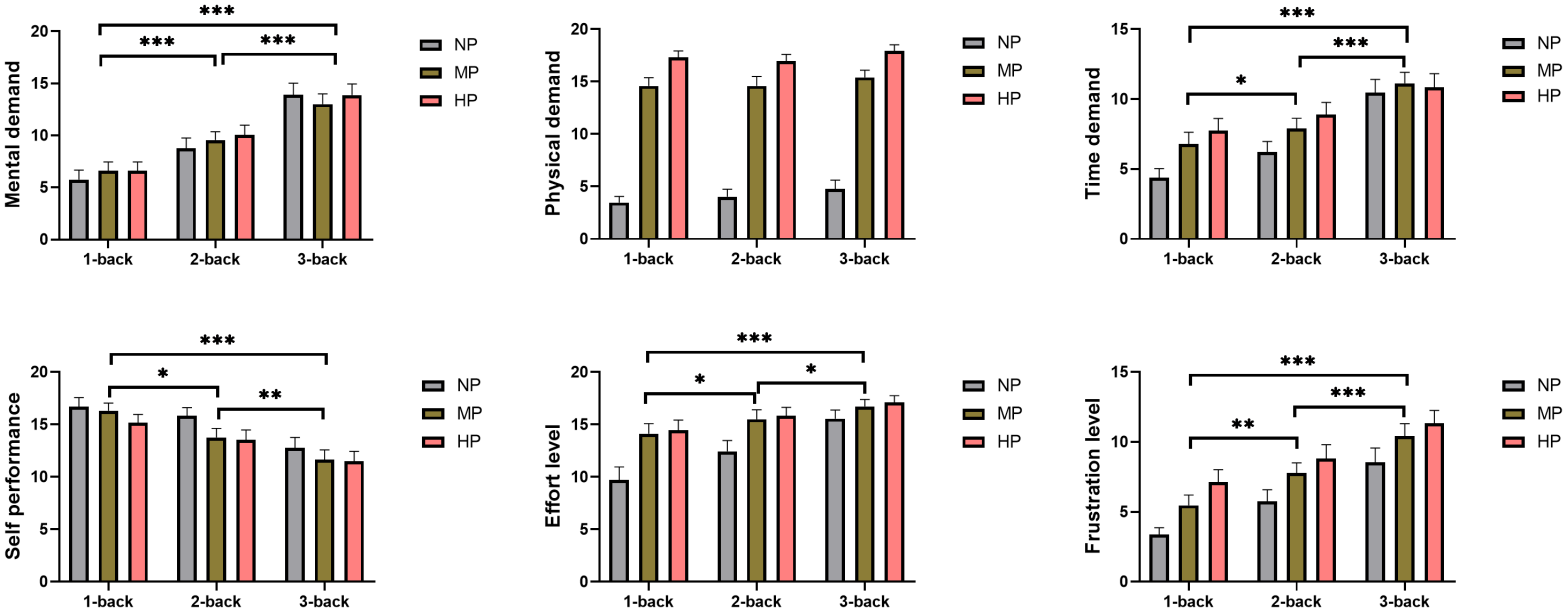
Main effect of cognitive and physical loads at different levels on National Aeronautics and Space Administration Task Load Index. *, **, and ***, respectively, represent main effects of cognitive load at *p* < 0.05, *p* < 0.01, and *p* < 0.001. HP, high physical load; MP, medium physical load; NP, none physical load.

Two-factor ANOVA for repeated measurement showed that PL and cognitive load had significant main effects, but no significant interaction effect on task-load perception of the NASA-TLX scale (Table 2). The post hoc test of cognitive load at different levels showed that, compared with 1-back, the scores of mental demand, time demand, effort level and frustration level increased significantly (*p* < 0.05), while self-performance decreased significantly (*p* < 0.05) under 2-back and 3-back conditions. Compared with 2-back, subjects’ subjective task-load was further increased under the 3-back condition (Figure 4). Furthermore, the main effects of PL at 3 levels showed that compared with NP, the subjective scores including physical demand (*P_MP_* < 0.001*, P_HP_* < 0.001), time demand (*P_MP_* = 0.026, *P_HP_* = 0.002), effort level (*P_MP_* < 0.001*, P_HP_* < 0.001), frustration level (*P_MP_* = 0.005, *P_HP_* < 0.001) were significantly increased under MP and the HP, while self-performance score (*P_HP_* = 0.018) decreased. There was no significant difference of task-load perception, with the exception of physical demand (*P_HP_* < 0.001), between the MP and HP.

**Table 2:** Main effect and interaction effect of cognitive and PLs on NASA-TLX scores (*F*-value).

Dimensions	Cognitive load	PL	Interaction effect
Mental demand	44.800^c^	0.323	0.306
Physical demand	1.518	293.198^c^	0.111
Time demand	22.546^c^	5.026^b^	0.874
Performance	15.061^c^	2.989	0.325
Effort level	12.412^c^	11.078^c^	1.035
Frustration level	24.350^c^	10.743^c^	0.107

b and c represent the statistical difference level at *p* < 0.01 and *p* < 0.001, respectively.

NASA-TLX, National Aeronautics and Space Administration Task Load Index; PL, physical load.

**Figure 5:**
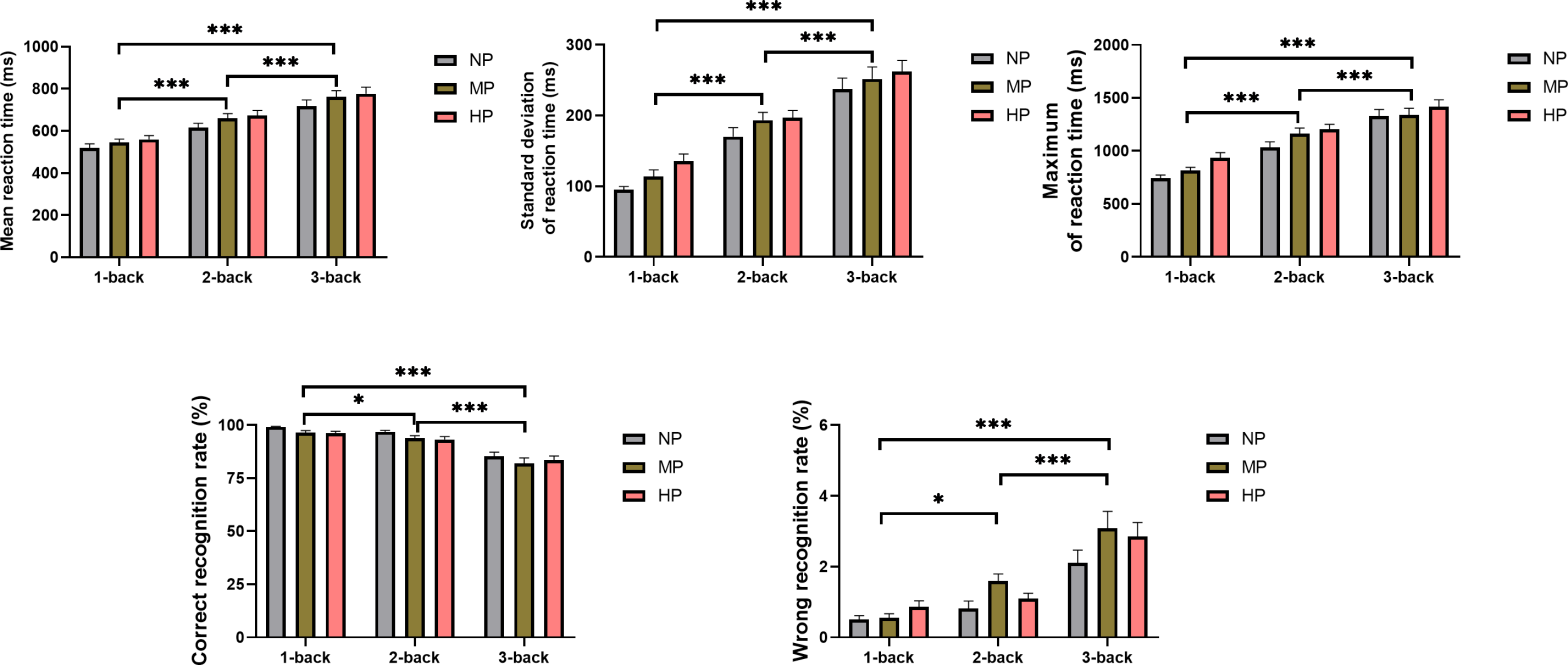
Main effect of cognitive and physical loads at different levels on task performance. * and *** represent main effects of cognitive load at *p* < 0.05 and *p* < 0.001, respectively. HP, high physical load; MP, medium physical load; NP, none physical load.

For task performance, the main effects of PL and cognitive load were significant, while the interaction effects were not significantly different (Table 3). The main effects of cognitive load revealed that with the increase in difficulty of the n-back task, that the average and maximal reaction times were prolonged, the standard deviation of the reaction time increased, the correct recognition rate decreased and the wrong recognition rate increased (Figure 5). Moreover, multiple comparisons of PLs at different levels showed that compared with the NP, the average reaction time (*P_MP_* = 0.048*, P_HP_* = 0.007), the maximal reaction time (*P_HP_* < 0.001) and the standard deviation of the reaction time (*P_Hp_* = 0.002) increased under MP and HP. The correct recognition rate (*P_MP_* = 0.013, *P_HP_* = 0.018) decreased, while the wrong recognition rate (*P_MP_* = 0.008, *P_Hp_* = 0.042) increased. The difference of task performance between MP and HP was not obvious. The findings that the task-load feelings of the subjects increased, and the task performance decreased suggested that n-back cognitive tasks induced different levels of MWL. And concurring PL during cognitive tasks would further increase the MWL level.

**Table 3:** Main effects and interaction effects of cognitive and PLs on task performance (*F*-value).

Task performance	Cognitive load	PL	Interaction effect
MRT	58.045^c^	3.924^a^	0.062
SDRT	90.316^c^	4.770^a^	0.191
MaxRT	80.336^c^	6.432^b^	0.500
CNR	73.439^c^	3.984^a^	0.164
WNR	43.525^c^	3.901^a^	1.030

a, b, and c represent the statistical difference level at *p* < 0.05, *p* < 0.01, and *p* < 0.001, respectively. CNR, the rate of correct number; MaxRT, maximum reaction time; MRT, mean reaction time; PL, physical load; SDRT, standard deviation of reaction time; WNR, rate of wrong number.

### 3.2 FC of PFC

#### 3.2.1 CORR-based function connectivity

**Figure 6:**
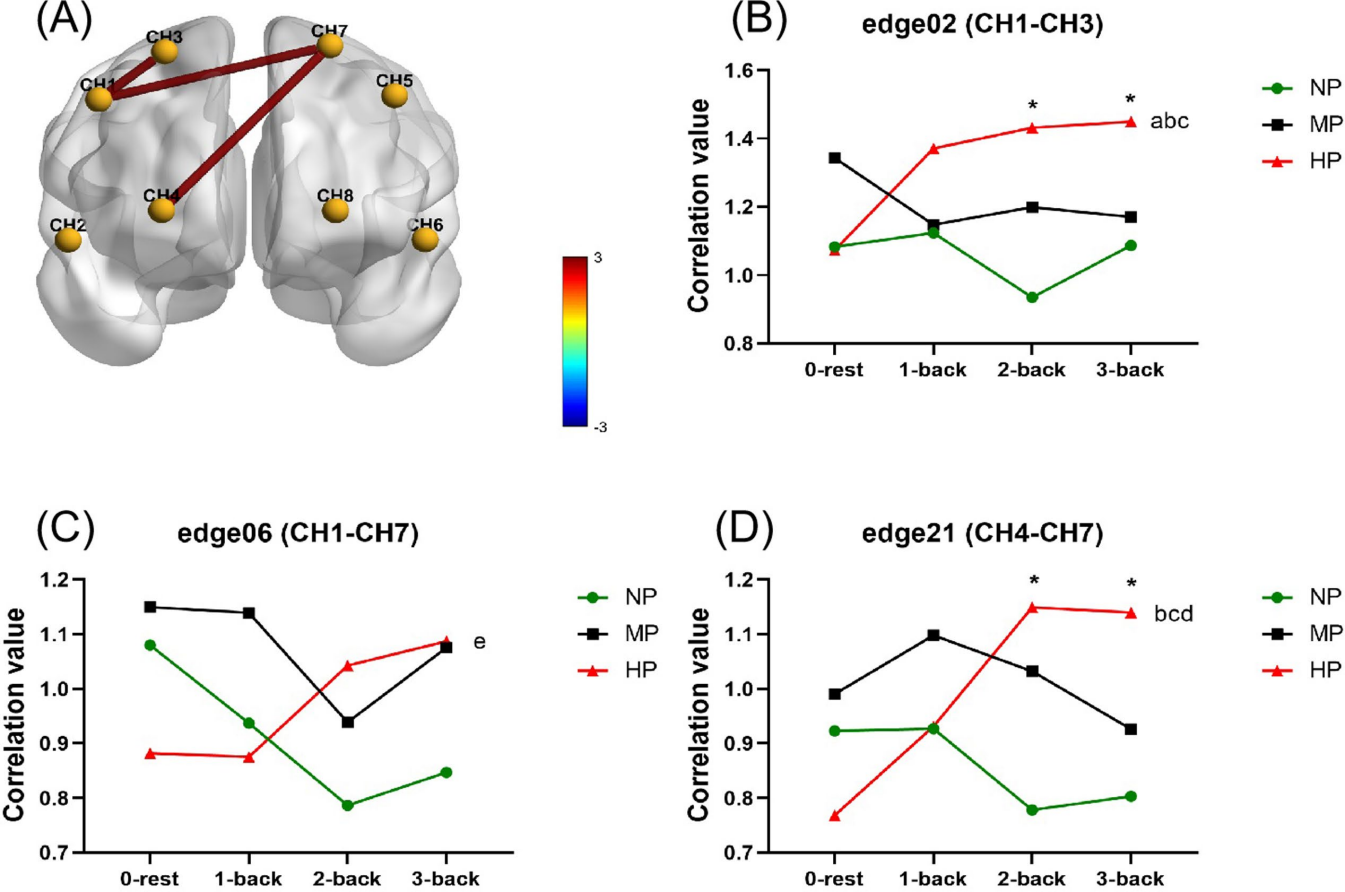
Statistical results of the correlation coefficient index at cognitive and physical levels. **(A)** Shows the significant interaction effects with legends of the *F*-value. **(B–D)** Shows the simple effects for edge 02 **(B)**, edge 06 **(C)** and edge 21 **(D)**. Specifically, * is for HP vs. NP at *p* < 0.05; a, b, c for 1-back, 2-back, 3-back vs. 0-rest at *p* < 0.05; d, e for 2-back and 3-back vs. 1-back at *p* < 0.05. H1, right dorsolateral prefrontal cortex; CH2, right ventrolateral prefrontal cortex; CH3 and CH4, right orbital frontal cortex; CH5, left dorsolateral prefrontal cortex; CH6, left ventrolateral prefrontal cortex; CH7 and CH8, left orbital frontal cortex; HP, high physical load; MP, medium physical load; NP, none physical load.

Under the influence of different levels of cognitive tasks and PL, the matrix and brain pilots of FC based on CORR index are shown in Supplementary Figures 1, 2. According to the results of repeated measurement ANOVA, cognitive tasks and PL had no significant main effects on the CORR value of each edge, while the interaction effects of the edge 02 (CH1-CH3), edge 06 (CH1-CH7) and edge 21 (CH4-CH7) was significant (*F_2_* = 3.361*, p* = 0.004; *F_6_* = 3.421, *p* = 0.004; *F_21_* = 3.635, *p* = 0.002) (Figure 6A). The *post hoc* test on the simple effects of PL showed that CORR values of edge 2 and edge 21 in the HP were significantly higher than those in the NP during 2-back task (*P_2_* < 0.001; *P_21_* = 0.008) and 3-back task (*P_2_* = 0.026, *P_21_* = 0.009). The simple effects of cognitive load showed that compared with the rest stage of the HP, the CORR values of edge 02 increased significantly during 1-back (*P* = 0.042), 2-back (*P* = 0.002) and 3-back (*p* = 0.012). For edge 06 under the HP, the CORR value during 1-back was significantly lower than that of 3-back (*p* = 0.038). Under the HP, the CORR value of edge 21 at rest stage was significantly lower than that of 2-back (*p* = 0.005) and 3-back (*p* = 0.001), and that of 1-back was significantly lower than that of 2-back (*p* = 0.033). There were no significant simple effects among other cognitive load and physical levels. The results demonstrated that the cognitive load increased, and the FC based on the CORR index at edge 02, edge 06 and edge 21 increased under the HP. The increase of PL also strengthened FC at edge 02 and 21 under higher cognitive load conditions.

#### 3.2.2 COH- based function connectivity

**Figure 7:**
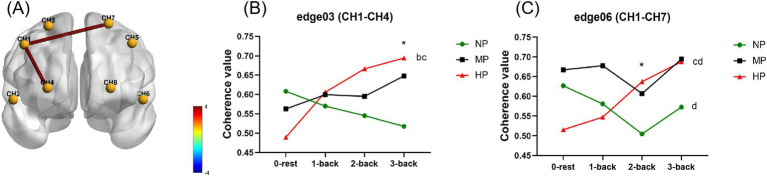
Statistical results of the coherence value index at cognitive and physical levels. **(A)** Shows the significant interaction effects with legends of the *F*-value. **(B,C)** Shows the simple effects for edge 03 **(B)** and edge 06 **(C)**. Specifically, * is for HP vs. NP at *p* < 0.05; b, c for 2-back and 3-back vs. 0-rest at *p* < 0.05; d is for 2-back vs. 1-back at *p* < 0.05. CH1, right dorsolateral prefrontal cortex; CH2, right ventrolateral prefrontal cortex; CH3 and CH4, right orbital frontal cortex; CH5, left dorsolateral prefrontal cortex; CH6, left ventrolateral prefrontal cortex; CH7 and CH8, left orbital frontal cortex; HP, high physical load; MP, medium physical load; NP, none physical load.

Under the influence of cognitive and PLs, the FC patterns of PFC based on COH index are shown in Supplementary Figure 3. Statistical results showed that cognitive load and PL had no significant main effects on the COH index of each edge, while the interaction effects at edge 03 (CH1-CH4) and edge 06 (CH1-CH7) were significantly different (*F_3_* = 4.328, *p* = 0.008; *F_6_* = 4.753*, p* = 0.006) (Figure 7A). The simple effects of PL showed that when performing the 2-back task, COH of edge 06 under the HP was significantly higher than under the NP (*p* = 0.035). For the 3-back task, COH of edge 03 at the HP level was significantly higher than that at the NP level (*p* = 0.008). Furthermore, the COH of edge-6 during 1-back was significantly higher than that during the 2-back task under the NP (*p* = 0.046). Compared to the rest stage, the COH of edge 03 was significantly increased during 2-back (*p* = 0.002) and 3-back (*p* < 0.001) at the HP level. For edge 06, the COH significantly increased during 3-back compared to that at the rest stage (*p* = 0.002), and this index during 2-back was also significantly higher than that of 1-back (*p* = 0.003). There were no significant simple effects among other conditions. The results showed that the FC based on the COH index at edge 03 and edge 06 increased with an increase in the cognitive load under a HP. And the HP also increased FC at edge 03 and edge 06 at the higher level of cognitive load.

#### 3.2.3 PLV-based function connectivity

**Figure 8:**
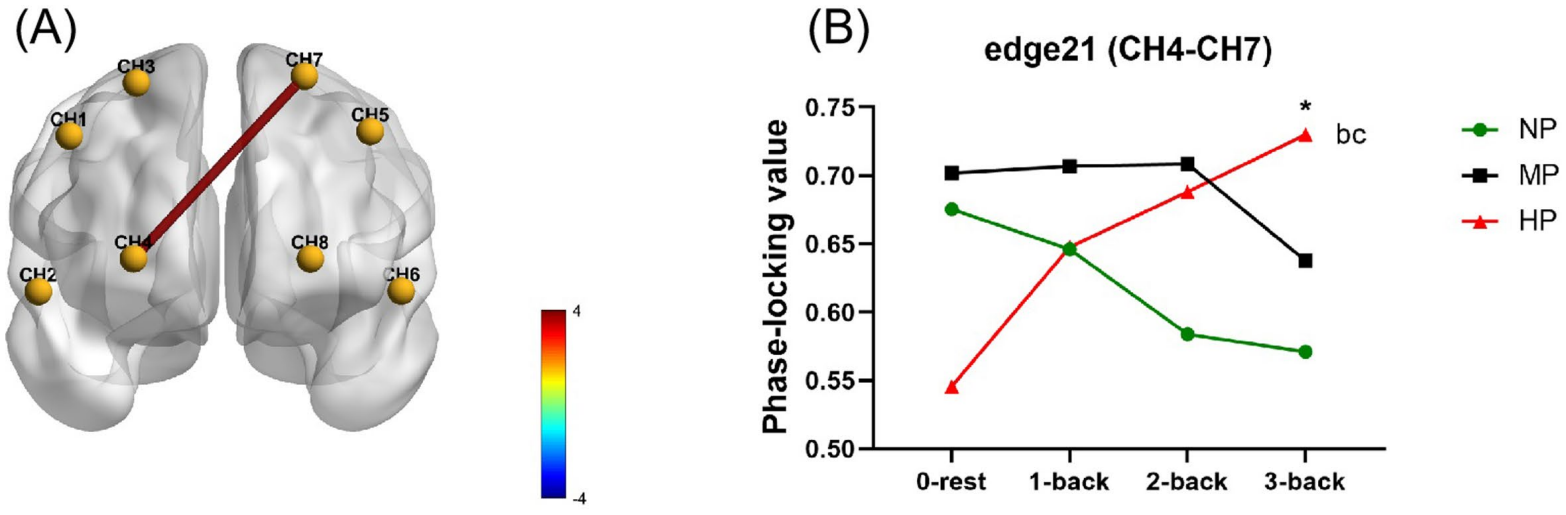
Statistical results of the phase-locking value index at cognitive and physical levels. **(A)** Shows the significant interaction effects with legends of the *F*-value. **(B)** Shows the simple effects for edge 21. Specifically, * is for HP vs. NP at *p* < 0.05; b, c for 2-back and 3-back vs. 0-rest at *p* < 0.05. CH1, right dorsolateral prefrontal cortex; CH2, right ventrolateral prefrontal cortex; CH3 and CH4, right orbital frontal cortex; CH5, left dorsolateral prefrontal cortex; CH6, left ventrolateral prefrontal cortex; CH7 and CH8, left orbital frontal cortex; HP, high physical load; MP, medium physical load; NP, none physical load.

Under the influence of cognitive load and PL, the PLV-based FC mode of each edge are shown in Supplementary Figure 4. It was clear that cognitive load and PL had no significant main effects, while the interaction effect on the PLV index of edge 21 (CH4-CH7) was significant (*F* = 4.529*, p* = 0.010) (Figure 8A). The simple effects of the *post hoc* test showed that PLV of edge 21 at the HP level was significantly higher than that at the NP level during the 3-back task (*p* = 0.004). Under the HP, PLV of edge 21 at the rest stage was significantly lower than that during 2-back (*p* = 0.019) and 3-back (*p* < 0.001). There were no significant simple effects among other conditions. The results showed that the PLV-based FC at edge 21 significantly increased line, with cognitive load increases at a HP level. When performing the 3-back task, PL also further increased FC strength in the PFC.

## 4 Discussion

This study explored MWL assessment from the perspective of actual work scenarios, and focused on the combined effects of physical and cognitive loads on behavioral and prefrontal hemodynamic features. Previous studies have shown that the actual work scene is different from the laboratory environment, and its consequences are also different. In the study of Liebherr et al., compared with quiet laboratory conditions, the accuracy of auditory Oddball tasks significantly decreased in two “real world” conditions (walking in the playground and navigation of campus), and the mismatch negative wave and P_300_ wave of event-related potential components were also different (Liebherr et al., 2021). Thus, it was of practical significance for actual mental work scenarios to integrate non-cognitive factors such as physical activities into the current research project. Presently, some cognitive tasks (n-back, stroop, etc.) are widely used to induce the MWL state in laboratory studies (Karthikeyan et al., 2021; Wriessnegger et al., 2021; Nikooharf Salehi et al., 2022). Based on such projects without non-cognitive factors, the neurophysiological features or evaluation models will cause some problems in matching between the training mode and practical application (Albuquerque et al., 2020). They may not be suitable for practical dynamic scenarios such as flying and driving. Researchers have gradually paid attention to the interaction effects between cognitive tasks and other physiological states on MWL assessment. For example, Zheng et al. combined the n-back working memory tasks with physical factors (bicycle riding) to study the activation of the cerebral cortex (Zheng et al., 2022). We previously studied the combined effects of cognitive load (n-back) and PL (isotonic contraction of the left upper limb) on autonomic activation based on heart rate variability (Cheng et al., 2024). This paper focuses on the impact on brain function. Compared to EEG and fMRI, fNIRS demonstrates better robustness to motion artifacts and environmental noise, while also being more user-friendly, making it well-suited for studying brain activity in real-world environments (Gateau et al., 2018). Therefore, this study utilized fNIRS. Previous studies on MWL or cognitive tasks have typically focused on the PFC. Causse et al. (2024) examined stress and MWL, while Liang et al. (2023) investigated the effects of environmental factors on MWL, highlighting corresponding changes in activation of the left and right DLPFC. Based on the literature, we selected these regions as the areas of interest for this study.

The calculation of COH, like Pearson’s CORR, followed the methods outlined in the study of Chao et al. (2021). In studies on EEG-based FC, such as (Lee et al., 2014), CORR and COH indicators were thoroughly described. For example, “In the early era of EEG research, correlation was most commonly used to investigate the similarity between two EEG signals, based on the assumption that a higher correlation map indicates a stronger relationship between two signals. Coherence gives similar information as correlation, but it includes the covariation between two signals as a function of frequency. According to another study on FC using fMRI (Novelli et al., 2024), “the Coherence model actually measured cross-spectral density, which is a second-order summary statistic of the time series data. This is closely related to Pearson’s correlation, another second-order statistic. These two factors often influence EEG signals; however, since near-infrared light is an optical signal rather than an electrical signal, it is theoretically unaffected by background coherence or volume conduction effects.

This study successfully induced different MWL states using the n-back working memory task, characterized by increased task-load perception, prolonged reaction time, and decreased task accuracy. The practice of inducing high cognitive workload with the n-back task has been frequently used in previous studies (Karthikeyan et al., 2021; Wriessnegger et al., 2021; Dallaway et al., 2022), and also proven by our previous research (Cheng et al., 2024). During the n-back tasks, adding physical activities can also affect the behavioral indicators of cognitive load. The results of our experiments suggested that concurrence PL further enhanced the effects of cognitive load. It should be noted that some other studies results seem to be inconsistent with those of our current study. These studies reported that response time of cognitive tasks was shortened under the influence of physical activities (Fujihara et al., 2021; Gao et al., 2021; Liu et al., 2023), such as cycling (Zheng et al., 2022) and resistance exercise (Engeroff et al., 2022). This reason has been also explained by our previous study that the differences in results may be caused by different experimental protocols (Cheng et al., 2024). Specifically, cognitive and physical tasks were performed simultaneously in the current study, while the cognitive tests were conducted after the physical activity in other studies.

FC analysis based on the HbO concentration revealed that with an increase in the cognitive load, the FC strength among some regions in the PFC increased, and would be further enhanced by a high PL. Previous studies have found that an increase in the MWL level based on the n-back tasks lead to an increase of HbO concentration in the PFC (Molteni et al., 2008; Molteni et al., 2012), indicating that the degree of activation of these brain regions had increased. It was also shown that increase in the degree of activation of the PFC was positively correlated with subjective load ratings (Li et al., 2022) and negatively correlated with work performance (Yan et al., 2024). These results would explain the reason for the FC enhancement in PFC to some extent. In addition, the present study also indicated that changes of FC strength mainly happened between CH1 and other channels (CH3/4/7). In other words, the FC between the right DLPFC and the OFC increased, which suggested that the right DLPFC may be a key area under the combined effects of physical and cognitive factors. Zhang reported a similar phenomenon, namely that the PFC was activated during implicit cognitive reappraisal, and acute exercise enhanced the activation of this region. Differently, this change happened in the left DLPFC and left OFC, which is reflective of the partial neural bases of implicit cognitive reappraisal (Zhang et al., 2021). While some studies also found that when performing acute aerobic exercise, n-back tasks induced a decline of activation of some brain regions, including the bilateral frontal pole area, DLPFC, the right premotor and secondary cortex (Zheng et al., 2022). One article reported that the HbO content in left DLPFC did not change immediately after high intensity intermittent exercise, and then decreased significantly after 10 min of post exercise (Zhu et al., 2021). These results suggest that the decrease in PFC activation may be related to a compensatory decrease after exercise. Apparently, it is different from the moment of fNIRS signal acquisition in our study in that it happened during physical activity.

The limitation of the present study was that the cognitive load level was relatively low, and we were unable to explore the effects of cognitive overload on the activation in the PFC during physical activity. Studies have shown that the relationship between cognitive load and the degree of activation of the PFC is not linear, but follows a quadratic or cubic function (Durantin et al., 2014; McKendrick and Harwood, 2019), and found that cognitive overload may lead to attenuation of activation in the PFC. From the overall increase of FC in the PFC found in the present study, we speculated that the strength of the combined effect of physical and cognitive loads was relatively low. Therefore, future studies should be designed under a higher MWL condition induced by higher cognitive task difficulty, which may provide new insights into the interaction effects between cognitive load and other factors.

In summary, different MWL states were induced by working memory tasks (n-back) with different levels of difficulty. The combined condition of cognitive and physical factors were designed using isotonic contraction aerobic exercise of the left upper limb. It was found that concurrent physical activities could further increase MWL levels on the basis of effects on cognitive tasks. The fNIRS-based results also verified that the FC between the right DLPFC and the bilateral OFC was significantly enhanced under high physical and cognitive loads. From the perspective of PFC activation, this study supports effects of physical factor on operators’ mental load. The results provide a reference for studies and practical application for the real-time (or online) assessment of the MWL level in the natural environment.

## Data Availability

The raw data supporting the conclusions of this article will be made available by the authors, without undue reservation.
