# Supplementary material for: Enhancement of prefrontal functional connectivity under the influence of concurrent physical load during mental tasks
Source: Front Hum Neurosci. 2024 Dec 24;18:1500470. doi: 10.3389/fnhum.2024.1500470 (PMC11703818; doi:10.3389/fnhum.2024.1500470)
Supplement: Supplementary file 1 [file Table_1.DOCX]

Supplementary Material

## Supplementary Figures


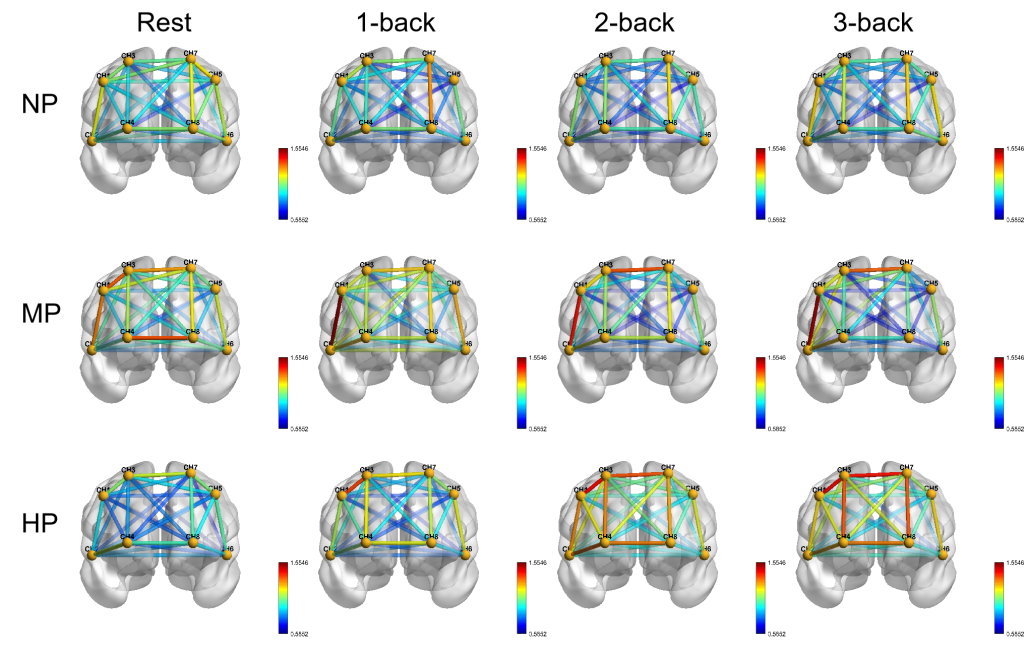


**Supplementary Figure 1.** Brain pilots of CORR-based FC at different cognitive and physical levels.

Note: CORR, correlation coefficient; FC, functional connectivity; HP, high physical load; MP, medium physical load; NP, none physical load.


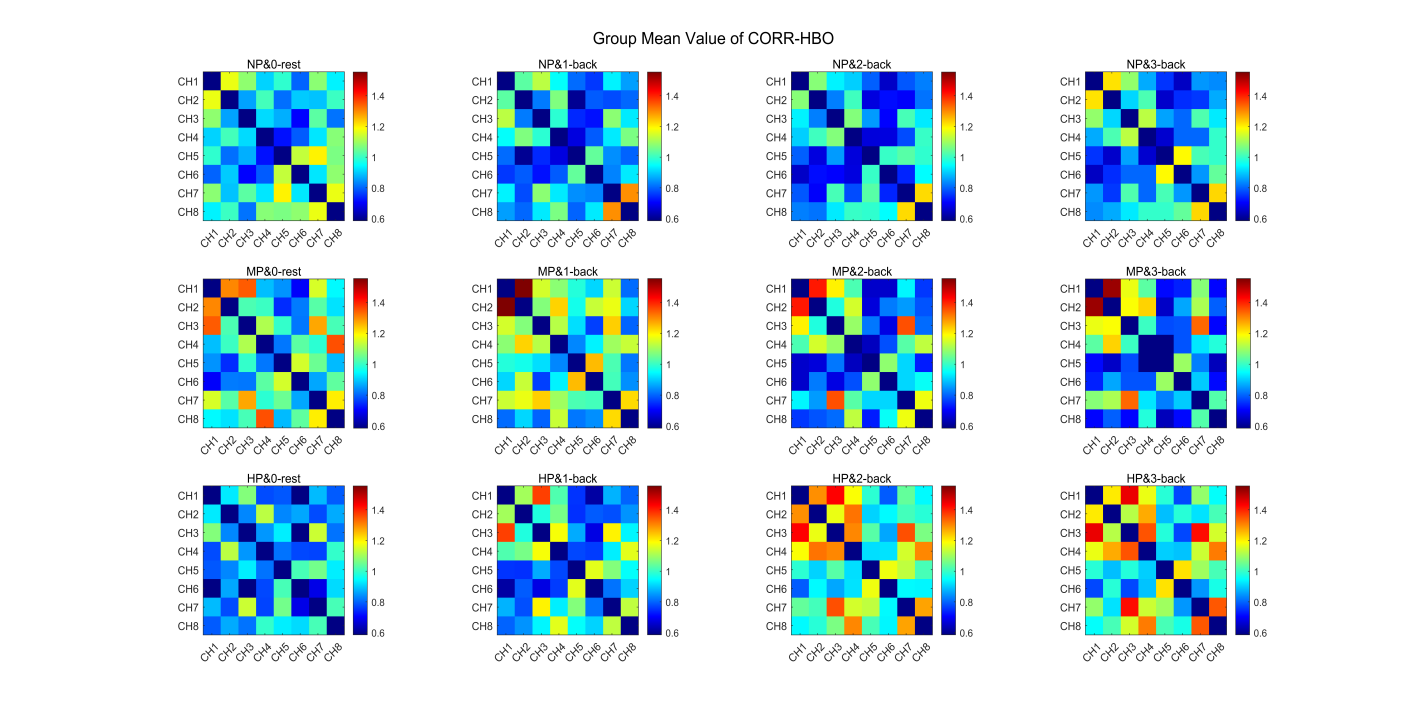


**Supplementary Figure 2.** Matrix pilots of FC based on the CORR index under different cognitive and physical levels.

Note: CORR, correlation coefficient; FC, functional connectivity; HP, high physical load; MP, medium physical load; NP, none physical load.


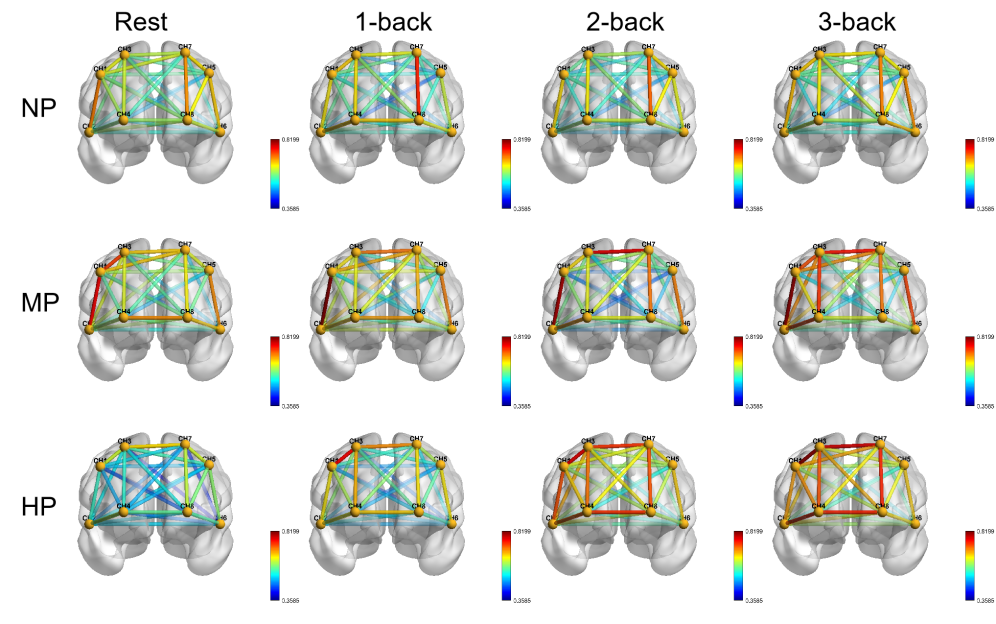


**Supplementary Figure 3.** COH-based FC at different cognitive and physical levels.

Note: COH, coherence value; FC, functional connectivity; HP, high physical load; MP, medium physical load; NP, none physical load.


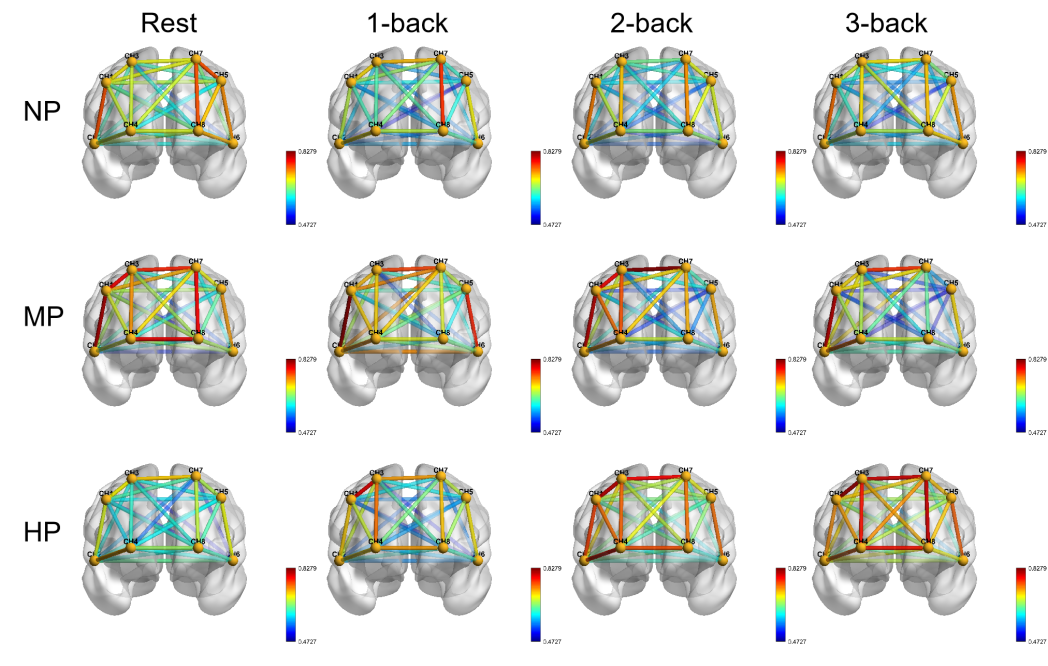


**Supplementary Figure 4.** PLV-based FC at different cognitive and physical levels.

Note: PLV, phase-locking value; FC, functional connectivity; HP, high physical load; MP, medium physical load; NP, none physical load.
